# Metabolic profiling of cholesterol and sex steroid hormones to monitor urological diseases

**DOI:** 10.1530/ERC-16-0285

**Published:** 2016-10-01

**Authors:** Ju-Yeon Moon, Man Ho Choi, Jayoung Kim

**Affiliations:** 1 Molecular Recognition Research CenterKorea Institute of Science and Technology, Seoul, Korea; 2 Departments of Surgery and Biomedical SciencesCedars-Sinai Medical Center, Los Angeles, California, USA; 3 Department of MedicineUniversity of California, Los Angeles, California, USA

**Keywords:** steroid, cholesterol, biomarkers, mass spectrometry, metabolomics, urological cancers, prostate diseases

## Abstract

Cholesterol and sex steroid hormones including androgens and estrogens play a critical role in the development and progression of urological diseases such as prostate cancer. This disease remains the most commonly diagnosed malignant tumor in men and is the leading cause of death from different cancers. Attempts to understand the role of cholesterol and steroid metabolism in urological diseases have been ongoing for many years, but despite this, our mechanistic and translational understanding remains elusive. In order to further evaluate the problem, we have taken an interest in metabolomics; a discipline dedicated to the systematic study of biologically active metabolites in cells, tissues, hair and biofluids. Recently, we provided evidence that a quantitative measurement of cholesterol and sex steroid metabolites can be successfully achieved using hair of human and mouse models. The overall goal of this short review article is to introduce current metabolomic technologies for the quantitative biomarker assay development and also to provide new insight into understanding the underlying mechanisms that trigger the pathological condition. Furthermore, this review will place a particular emphasis on how to prepare biospecimens (e.g., hair fiber), quantify molecular profiles and assess their clinical significance in various urological diseases.

## Introduction

Urological health conditions have become increasingly prevalent in the world, affecting individuals spanning a multitude of ages. Prostate cancer (PC) is one such condition that has seen an exponential rise in the number of cases, with over 220,000 new cases having been recorded in 2015 alone. The effects of PC can even be seen outside of the USA, as it is the most common cancer among men in all developed countries. However, not all urological health problems are related to cancer, and the most common of these include the common occurrence of urinary tract infection, kidney stones, incontinence and benign prostatic hyperplasia (BPH). These diseases pose both a financial and physiological burden, indicating the need for further research in the prevention and study of these urological conditions. The use of biomarkers for early diagnosis in patients would be valuable in reducing the recurrence and progression of these urological health problems. As an example, there are clinical needs for biomarkers to identify PC patients who have aggressive disease and are more likely to experience disease progression, which could help increase the ability to manage patients with urological disease.

Research has indicated that high levels of cholesterol and sex steroid hormones are risk factors of urological disease (Allott & Hursting 2015). A correlation has been studied, relating a typical Western diet to an increase in the risk of urological disease (Ito 2014, YuPeng *et al*. 2015). Western diet normally features a high intake of red meat and dairy products, providing a high intake of cholesterol and calories to individuals consuming such foods (Ito 2014, YuPeng *et al*. 2015). These studies have also provided evidence that patients with a metabolic syndrome (e.g., obesity, impaired fasting glucose tolerance, high blood pressure, hypertension, dyslipidemia, type 2 diabetes and cardiovascular diseases) were more likely to have great prostate volume increase (Gacci *et al*. 2015), suggesting a high concentration of cholesterol within urological disease. Hypercholesterolemia, an obesity-associated co-morbidity, influences approximately 20% of the US population (Fryar *et al*. 2010). Furthermore, cholesterol-lowering drugs such as statins may reduce the risk of PC (Nielsen *et al*. 2012).

Sex steroids and their receptors play a crucial role in the determination of urological disease development. Androgen and its derivatives including dihydrotestosterone (DHT) are vital in not only male development but also the development of PC and BPH. Following androgen binding, androgen receptors undergo a multi-step process involving dimerization, phosphorylation and translocation to the nucleus. Once localized, the receptor acts as a transcription factor and binds to androgen receptor elements (AREs) in order to begin assembling a transcription complex of co-activators and co-repressors (Dehm & Tindall 2007). These complexes are key oncogenic risk factors associated with the increased risk of PC and BPH.

In order to better understand the metabolism and internal mechanisms underlying urological diseases, several resources have been studied. Metabolomic fingerprints have been analyzed in addition to the use of non-invasive biomarkers such as urine or blood-based assays. Hair-based metabolomic profiles could be useful in confirming the correlation between cholesterol and sex steroid hormones with urological diseases. Using human and animal hair samples in order to study the metabolite process specific to urological disease, our research group established the novel mass spectrometry-based protocols for steroid metabolomics with the goal of monitoring hormone levels, which can be used for drug treatment of PC and BPH patients.

This short review article aims to provide support for the claim that a correlation exists between cholesterol and steroid sex hormones with urological diseases. By specifically examining PC and BHP, we discuss the significance of cholesterol and steroid sex hormones associated with these health conditions, while also introducing current technologies that can be used to measure the amount of cholesterol and sex steroid hormones in various sources throughout the body (tissues, urine, blood and hair). The final topic deals with the use of hair metabolomics to identify potential biomarkers for PC and BPH.

## Metabolism of cholesterol and sex steroid hormones

**Figure 1:**
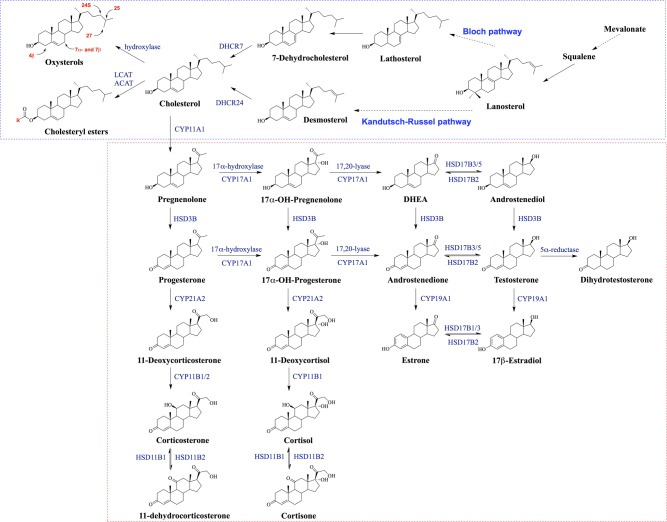
Overview of cholesterol and steroid hormone pathways.

Cholesterol is a crucial component of mammalian cell membranes, as it serves diverse cellular functions – including the modulation of membrane permeability and fluidity (Maxfield & Tabas 2005). Cholesterol synthesis pathways are shown in Fig. 1. Cholesterol is also the precursor of all steroid hormones and bile acids and plays important roles in membrane trafficking, transmembrane signaling processes as well as cell proliferation (Goedeke & Fernandez-Hernando 2012). Cholesterol is made from the conversion of citrate, derived from the tricarboxylic acid (TCA) cycle in the mitochondria. Here, acetyl coenzyme A (acetyl-CoA) is formed and followed by the mevalonate pathway. This combination of reactions is primarily regulated by a rate-limiting step catalyzed by 3-hydroxy-3-methylglutaryl-coenzyme A (HMG-CoA) reductase, an integral membrane protein of the smooth endoplasmic reticulum, which converts HMG-CoA to a six-carbon intermediate mevalonate. This intermediate is then metabolized via a series of isoprenoid intermediates to squalene (the polymerization of six five-carbon isoprene units to form the 30-carbon linear structure of squalene). The cyclization of squalene reacted by squalene cyclase and one molecule of O_2_ forms the four fused rings of the steroid nucleus, which results in the synthesis of lanosterol as a cholesterol precursor.

Cholesterol in tissues and blood is metabolized as follows. First, it can be fatty acylated to form cholesteryl esters (CEs) through sterol *O*-acyltransferase (also called acyl-CoA cholesterol acyltransferase or simple ACAT) or lecithin-cholesterol acyltransferase (LCAT, also called phosphatidylcholine-sterol *O*-acyltransferase). The CEs then serve as a major form of transporter as plasma lipoproteins, or as storage units in the form of lipid droplets (Kraemer 2007). Secondly, cholesterol can be oxidized to form oxygenated derivatives of cholesterol, termed oxysterols, by enzymatic (hydroxylase, cytochrome P450 (CYP) families) or non-enzymatic hydroxylations at the C-4, C-7, C-19, C-20, C-24, C-25 and C-27 positions (Pikuleva 2006, Son *et al*. 2014, Seo & Choi 2015). Oxysterols serve as regulators of cholesterol homeostasis, allowing cells to manage large cholesterol loads rapidly and avoid triggering cytotoxic events (Bjorkhem 2002, Bielska *et al*. 2012). For example, cholesterol activates 7α-hydroxylase (CYP7A1), which produces 7α-hydroxycholesterol as the major pathway for elimination of cholesterol from the body (Bjorkhem 2002). In addition, the most important oxysterols (as transport forms of cholesterol) are side chain-oxidized oxysterols at the C-24 or 27 position by CYP46 and CYP27, respectively, which flow continuously from peripheral tissues to the liver and become further oxidized into bile acids or other water-soluble metabolites (Nielsen *et al*. 2012). 4β-hydroxycholesterol catalyzed by CYP 3A4 may indicate slow elimination when its levels are high in the blood (Bodin *et al*. 2002). In addition, the 25-hydroxycholesterol that is produced and secreted by macrophages can regulate interleukin-1β, a potent cytokine, facilitating cross talk between cholesterol metabolism and the immune system (Simon 2014).

Cholesterol is also metabolized to steroid hormones, which regulate physiological and pharmacological processes in the body (Falkenstein *et al*. 2000). Steroidogenic enzymes are responsible for the biosynthesis of cholesterol from various steroid hormones including corticoids, progestins, androgens and estrogens. These are generally synthesized in the adrenal cortex, gonads (testes and ovaries), brain, placenta and adipose tissue (Falkenstein *et al*. 2000, Payne & Hales 2004). In biosynthetic pathways of steroid hormones, two major types of enzymes are involved: cytochrome P450 enzymes (CYPs) and hydroxysteroid dehydrogenases (HSDs). Initially, steroid hormones start with the conversion of cholesterol to pregnenolone by rate-limiting enzyme CYP11A (cholesterol side-chain cleavage), which is bound to the inner membrane of the mitochondrion in all steroidogenic tissues. This acute regulation is mediated by the steroidogenic acute regulatory protein (StAR) on the outer membrane, which facilitates the rapid influx of cholesterol into mitochondria (Payne & Hales 2004, Miller 2008).

## Cholesterol and sex steroid hormones in prostate health

Cholesterol, a critical component of the cellular plasma membrane, contributes to the maintenance of plasma membrane fluidity. Also, cholesterol is an important component of lipid raft micro-domains on plasma membrane and regulates intracellular signaling processes (Krycer & Brown 2013). Cholesterol is also the precursor for endogenous sex steroid biosynthesis, suggesting that elevated serum cholesterol levels might be somehow linked to the increased risk of prostate cancer (Fagherazzi *et al*. 2010). Steroid biosynthesis may be an important mechanism linking cholesterol and prostate cancer and BPH.

StAR and CYP11A are involved in converting cholesterol into pregnenolone and progesterone, which are sequentially converted to DHEA and androstenedione by CYP17A. DHEA is then converted to form testosterone and then DHT via HSD3B, HSD17B3 (or AKR1C3) and SRD5A. The 5α-androstanedione pathway leads to produce DHEA, androstenedione and then testosterone. There are multiple enzymes, which actively play roles in cholesterol and sex steroid hormone synthesis. AKR1C1 converts DHT to 5α-androstane-3,17-diol (3α-androstanediol or 3α-diol) and AKR1C2 converts DHT to 5α-androstane-3,17-diol (3β-diol). UGT2B15 and UTG2B17 irreversibly inhibit androgen signaling by glucuronidation, which is the known rate-limiting step of androgen signaling.

Prostate epithelial cells have higher cholesterol content, compared with other organs, and cholesterol levels increase during progression of normal healthy prostate into PC or BPH (Krycer & Brown 2013), suggesting that cholesterol accumulation may benefit prostate cancer or BPH progression. Accumulating evidence demonstrates that elevated cholesterol is a risk factor of more aggressive PC – in terms of recurrence or mortality (Platz *et al*. 2008, 2009, Farwell *et al*. 2011, Mondul *et al*. 2011, Shafique *et al*. 2012). Our previous studies also support the hypothesis that cholesterol promotes PC growth *in vitro* and *in vivo* (Zhuang *et al*. 2002). Cholesterol-lowering drugs (e.g., statins, zetia or combination of both) also lowered serum as well as intratumoral androgen levels, leading to the arrest of tumor growth (Mostaghel *et al*. 2012). Statins have been used for patients with cardiovascular diseases (Ridker & Cook 2013). There are a series of epidemiological studies suggesting that statins could reduce cancer risk (Farwell *et al*. 2008), chronic inflammation and angiogenesis (Demierre *et al*. 2005, Pelton *et al*. 2012). However, several meta-analyses have also reported a null association between statin use and risk of prostate cancer recurrence (Mass *et al*. 2012, Park *et al*. 2013, Scosyrev *et al*. 2013), suggesting that they remain contradictory in the field.

## Analytical techniques for sex steroid metabolome

### Tissue- and biofluid-based metabolite profiling

As the most common specimens used in biomarker discovery, tissues and biological fluids have been used for metabolite profiling. Both formalin-fixed, paraffin-embedded (FFPE) and fresh-frozen tissue specimens can be used for the tissue-based metabolomic studies. Metabolite extraction from FFPE tissues includes de-paraffinization steps with xylene, homogenization in MeOH:H_2_O solution (1:1 v/v), vortexing and sonication.

Biofluids such as urine and serum have a great advantage of being the easiest samples to work with, urine being the most common and accessible samples for metabolomic analyses. Metabolome in urine can be greatly influenced by age, occupation, environmental factors, different diets, hormones and lifestyle such as exercise, and urine specimens should be immediately stored within a few hours after sample collection at –80°C until further analysis.

In order to identify the steroid signatures and to suggest steroid metabolism-associated enzyme activities through profiling of tissues or biofluid-derived metabolites, we can use quantitative mass spectrometry combined with gas or liquid chromatographic separation techniques (GC–MS or LC–MS) for steroid profiling. Our previous studies to develop the quantitative steroid signatures using GC–MS (Ha *et al*. 2009, Moon *et al*. 2009) demonstrated that we could measure concentrations of over 65 endogenous steroids and cholesterols in plasma or urine samples at a time. In addition, LC–MS-based steroid profiling enables to quantify 21 endogenous corticoids including urinary glucocorticoids and mineralocorticoids (Cho *et al*. 2009). Both GC–MS and LC–MS urinary steroid signatures were applied into the samples obtained from patients with PC or BPH from age- and gender-matched healthy subjects.

**Figure 2:**
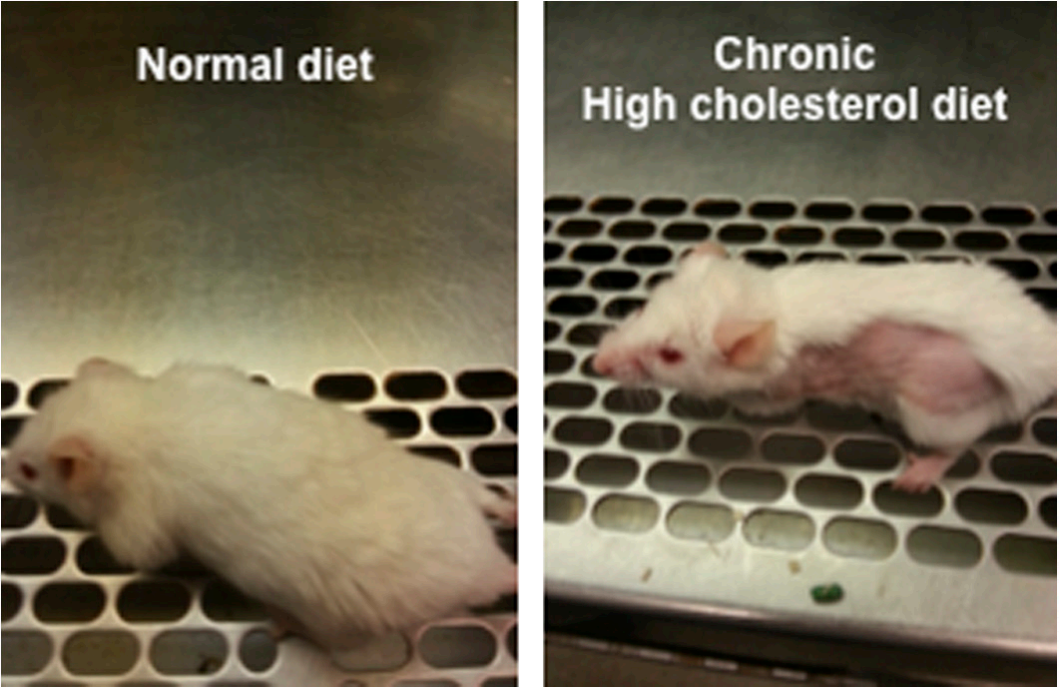
Our metabolomic profile showing the distinct patterns between prostate cancer and healthy controls.

As a good example, our GC–MS analysis data showed that urinary cholesterol levels in BPH patients were significantly increased in those with healthy controls (*P* = 0.015) (Fig. 2A). First morning urine samples obtained from 59 BPH patients (age: 65.3 ± 8.2 years) and 41 healthy male subjects (age: 56.7 ± 7.1 years) were used for this analysis. We screened various cholesterol and sex hormones and found that cholesterol level could segregate BPH patients from healthy controls (AUC, 0.66) (Fig. 2B).

### Hair metabolomics for hair-based metabolite profile

Although urine and blood are widely used to measure sex steroid hormones in many clinical and toxicological studies (Wudy *et al*. 2000, Choi *et al*. 2011, Choi & Chung 2015, Son *et al*. 2015), their concentrations fluctuate on a daily basis. Additionally, careful handling of biological fluids is required for sample collection, handling, processing, storage and transport to the laboratory (Tworoger & Hankinson 2006) under controlled circumstances. In contrast, hair grows about 1 cm per month (Wennig 2000). Thus, it can offer the possibility of reflecting and revealing historical information of exposure to drug abuse, environmental toxins and endogenous or exogenous hormones over several months (Villain *et al*. 2004). Moreover, hair sampling is non-invasive and the storage and processing of it is much simpler than plasma or serum (Sulek *et al*. 2014). Therefore, hair analysis has been used in chemical toxicology, forensic science, doping control and clinical applications (Cho *et al*. 2010, Pichini *et al*. 2012, Choi *et al*. 2013, Gray *et al*. 2013, Pereg *et al*. 2013, Chan *et al*. 2014, Veldhorst *et al*. 2014, Son *et al*. 2016).

#### Sample preparation: extraction from hair fiber

Hair analysis in steroid research is mainly coupled with solubilization or digestion of the hair matrix after cutting (Choi *et al*. 2001*a*, 2013, Cho *et al*. 2010). In general, androgens and sterols are extracted from the hair matrix by alkaline hydrolysis involving the complete digestion of hair (Choi & Chung 1999, Choi *et al*. 2000, Ryu *et al*. 2006), whereas corticoids are unstable under these conditions. Hence, ultrasonication with an organic solvent was also tested for steroid profiling including androgens, sterols, corticoids and progestins, which enables the profiling of hair steroids (Jung *et al*. 2011). A mixed solvent of methanol and dichloromethane is an effective solvent for lipid extraction from biological samples (Folch *et al*. 1957). However, absolute methanol was chosen as the extraction solvent to decrease sample complexity and simplify the sample purification steps. The hair strands were washed with isopropyl alcohol to prevent contamination and were then obtained simply by cutting the specific lengths from the proximal part of the vertex scalp (Jung *et al*. 2011). Thirty milligrams of chopped hair were incubated with 0.5 mL methanol in an ultrasonic bath for 1 h at 50°C. After cooling to room temperature, a methanolic solution was diluted with 5.5 mL sodium acetate buffer (pH 5.2) to less than 8% methanol. The samples were loaded directly onto the Oasis HLB^TM^ (divinylbenzene and *N*-vinylpyrrolidone) solid-phase extraction (SPE) cartridge, which is preferable for the sample purification of steroidal compounds (Moon *et al*. 2009, Choi & Chung 2015). The SPE procedure removed more effective interference from the hair matrix and gave a lower matrix background than liquid–liquid extraction (LLE) (Mondul *et al*. 2011). However, these extraction processes require a relatively large amount of hair matrix and extensive, time-consuming pretreatment procedures.

Recently, the pulverization method has been used to highly disintegrate hair components and has allowed for efficient extraction (Miyaguchi *et al*. 2007, Kim *et al*. 2011, Son *et al*. 2016). Compared with our previous techniques (Ryu *et al*. 2006, Jung *et al*. 2011), this method enables increased extractable surface area of the hair matrix through the destruction of the cuticle layer and thereby the permeation of an extraction solvent into the hair. The pulverization of hair using a ball mill such as zirconia beads was achieved for improved extraction yields of steroids and sterols and therefore can reduce sample preparation times as well as sample amounts significantly. Therefore, hair steroid analysis has been successfully applied in clinical applications using sampling of 100–150 strands of hair and extraction from a minimum of 10 mg (Choi *et al*. 2013). Hair sterols were measured in only two strands of 3 cm hair segments, corresponding roughly to a period of recent 3 months.

The sample preparation technique is also required for the removal of endogenous matrix components from lipid-rich samples. In particular, phospholipids are extremely abundant in hair as well as blood (Singh & Gershbein 1967). The use of hybrid precipitation/SPE plates for selective removal of phospholipids and precipitated proteins has been increasing over the past few years (Bylda *et al*. 2014). The hybrid SPE-precipitation cartridge (H-PPT) applies to reduce the phospholipid-based matrix effect, which is a superior purification method for sterol analysis (Pucci *et al*. 2009, Son *et al*. 2014) relative to membrane filtration (Miyaguchi *et al*. 2007). The H-PPT specifically retains phospholipids by Lewis acid–base interactions between the zirconia-coated silica particles bonded to the stationary phase and the phosphate group of the phospholipids, which provides simple yet rapid selective removal of interference (Bylda *et al*. 2014). When the extraction recoveries of the sterols were compared at different pulverization times (1, 2, 5, 10, 15 and 20 min) and different frequencies (10, 15, 20, 25 and 30 Hz), 10 min at 25 Hz was chosen as the optimized extraction method (Son *et al*. 2016). To facilitate sterol extraction, two strands of 3 cm-long hair samples were pulverized in 0.5 mL methanol using a TissueLyser for 10 min at 25 Hz in a 2 mL Safe-Lock tube containing three zirconia beads (3.0 mm I.D.). Bead-assisted liquid–liquid extraction via the addition of methanol and then centrifugation can be achieved simultaneously with pulverization, extraction and protein precipitation (Son *et al*. 2016). Samples were then loaded into H-PPT cartridges and eluted three times with 0.5 mL methanol. The matrix background such as proteins and phospholipids was easily removed and finally hair sterols were collected.

#### Sample pretreatment: chemical derivatization

In GC separation, derivatization of steroid molecules is a prerequisite step to generate compounds with better volatility, thermal stability and thereby improved chromatographic properties (Marcos & Pozo 2015). The common reactions used in GC analysis are silylation, acylation and alkylation (Choi & Chung 2015), depending on the individual properties of the steroid and the detection system. Silylation is the most widely used derivatization reaction in steroid analysis, and trimethylsilyl (TMS) derivatization is extensively used for most functional groups on steroid backbone, including aliphatic and phenolic alcohols, and carbonyl and amine groups. The purpose of this is to increase volatility as well as MS characteristics for GC–MS (Choi *et al*. 2002, Moon *et al*. 2009, Marcos & Pozo 2015). The most common reagents are *N*,*O*-bis (trimethylsilyl)-trifluoroacetamide (BSTFA) and the more volatile *N*-methyl-*N*-trimethylsilyltrifluoroacetamide (MSTFA) as a powerful trimethylsilyl (TMS) donor in the derivatization procedure (Shareef *et al*. 2006, Marcos & Pozo 2015). One of the most reported derivatization techniques in steroid profiling is the application of a mixture of MSTFA/ammonium iodide (NH_4_I)/dithioerythritol (DTE) in a ratio of 500:4:2 (v/w/w) (Moon *et al*. 2009, 2010, Jung *et al*. 2011, Son *et al*. 2015). Most steroids were monitored using their molecular ions as base peaks.

#### For the profiling of 18 sterols, including cholesterol, six CEs, three cholesterol precursors and eight OHCs

Cholesterol and cholesterol precursors have a hydroxyl group at the C-3 position, and OHCs have two polar functional groups: one is a hydroxyl group at the C-3 position and the other is a hydroxyl or ketone group at the C-4, C-7, C-19, C-20, C-24, C-25 or C-27 position. In TMS derivatization, both hydroxyl and carbonyl ketone groups were derivatized with TMS, whereas CEs were unaffected by TMS agents because they do not have polar groups in their chemical structures. The characteristic ions of cholesterol were observed at *m/z* 458 [M]^+^, *m/z* 443 [M–15; M–CH_3_]^+^, *m/z* 368 [M–90; M–OTMS]^+^, *m/z* 353 [M–90–15; M–OTMS–CH_3_]^+^, *m/z* 329 [M–129; M–TMS-O^+ ^= CHCH = CH_2_]^+^ and *m/z* 129 [TMS-O^+ ^= CHCH = CH_2_]^+^, which are in accordance with a general mass spectral interpretation. Among these fragments, the *m/z* 368 ion was chosen as the quantitative ion. All CEs generated a base peak at *m/z* 368 by cleavage of the ester bond, regardless of the fatty acid moiety (Jung *et al*. 2009). The quantitative ion of desmosterol was selected to be the *m/z* 343 ion that was formed by the loss of the side chain and two nuclear hydrogens. The quantitative ions of lathosterol and lanosterol were selected to be *m/z* 458 [M]^+^ and *m/z* 393 [M–90–15]^+^, respectively. In addition, OHCs showed different fragmentation patterns depending on the –OH positions (Moon *et al*. 2014). These results may provide useful information about the chemical structures of cholesterol and its metabolites.

#### For chemical transformation of multi-functional steroids

Mixed derivatization is performed to improve physical and chemical properties and mass spectral characteristics. Sensitive and selective quantification of eight steroids related to androgen biosynthesis in human hair was achieved by a combination of TMS and pentafluorophenyldimethylsilylation (flophemesyl-TMS) (Choi & Chung 1999). The spectra of flophemesyl derivatives generally display intense molecular ions under electron impact ionization, resulting in enhanced chromatographic selectivity and mass spectral information with sensitive detection (Choi *et al*. 2001*a*, Choi & Chung 2015). Recently, the enhanced GC–MS analytical selectivity and sensitivity were allowed for quantitative analysis of estrogen metabolites in urine samples obtained from the postmenopausal female patients with osteoporosis (Moon *et al*. 2011*b*). It was successfully achieved by two-phase extractive ethoxycarbonylation (EOC) and subsequent pentafluoropropionyl (PFP) derivatization. In case of estrogen profiling, the ultra-sensitive LC–MS analytical method has been conducted with a novel chemical derivatization procedure, which formed analytes as pre-ionized *N*-methyl pyridinium-3-sulfonyl (NMPS) derivatives (Wang *et al*. 2015).

#### Analytical instrumentation

Although radio­immunoassay (RIA) or enzyme immunoassays (EIA) are widely used to evaluate the quantification of steroid molecules (Thomson *et al*. 2009, Musshoff *et al*. 2012, Chan *et al*. 2014), the specificity of these methods is relatively low, which may result in an overestimation of the actual steroid content in samples. Furthermore, only single enzymes are estimated at a single time (Spiehler 2000, Hsing *et al*. 2007, Wood *et al*. 2008), making the method even more inaccurate. In contrast, mass spectrometry-based quantification has better reproducibility and generates multi-targeted profiling analysis (Cho *et al*. 2009, Ha *et al*. 2009, Jung *et al*. 2009, 2011, Moon *et al*. 2009, Son *et al*. 2015).

Several mass spectrometric methods for the measurement of steroids and sterols from various biological matrices have been proposed, coupled to GC (Ahmida *et al*. 2006, Ryu *et al*. 2006, Moon *et al*. 2009, 2011b 2014, Choi *et al*. 2011, 2013, Son *et al*. 2014, 2015) or LC (Lembcke *et al*. 2005, Griffiths *et al*. 2008, DeBarber *et al*. 2008, Honda *et al*. 2009, Karu *et al*. 2011). The LC–MS methods based on electrospray and atmospheric pressure chemical ionization techniques have been conducted with a good sensitivity and chromatographic resolution of estrogens (Falk *et al*. 2008, Penning *et al*. 2010, Wang *et al*. 2015), sterols and oxysterols (Burkard *et al*. 2004, Karuna *et al*. 2009, McDonald *et al*. 2012). However, the method often requires sample derivatization with dansyl chloride (Falk *et al*. 2008), pentafluorobenzyl chloride (Penning *et al*. 2010), Girard P hydrazine (Griffiths *et al*. 2006), picolinyl esterification (Yamashita *et al*. 2007, Honda *et al*. 2009) and NMPS (Wang *et al*. 2015) to improve ionization efficiencies and detection sensitivity. These methods enable to quantify the analytes in the low pg/mL ranges but are time-consuming because they require derivatization (Yamashita *et al*. 2007, Honda *et al*. 2009) and a long analytical run (Xu *et al*. 2007). Girard P derivatization can be seen in more detailed structure information due to MS^3^ (MS/MS/MS) applicability, but it appeared more laborious than GC–MS-based methods (Griffiths *et al*. 2006).

In particular, GC–MS with electron impact ionization is used widely for the measurement of steroid hormones with good analytical efficiencies as well as structural information. Initially, eight steroids related to androgen biosynthesis and two main estrogens (estrone and 17β-estradiol) were determined in hair (Choi *et al*. 2000, Choi & Chung 2015). In 2011, the simultaneous quantification of hair steroids, including androgens, progestins, corticoids and sterols by GC–MS method in selected ion monitoring (SIM) mode, was successfully validated to evaluate the concentrations of individual steroids as well as the activities of the enzymes responsible for steroidogenesis in hair follicles and sebaceous glands (Jung *et al*. 2011). This can synthesize many steroids from cholesterol or locally convert circulating steroids with a range of metabolic enzymes (Chen *et al*. 2002, Ohnemus *et al*. 2006). For hair steroid profiling, 62 steroids were analyzed on an Ultra-1 capillary column (25 m × 0.2 mm i.d., 0.33 μm film thickness), and only 20 hair steroids, including eight androgens, three progestins, five sterols and four corticoids, were detectable (Jung *et al*. 2011).

Compared with the conventional GC–MS techniques using a fused silica capillary column (Ahmida *et al*. 2006), high-temperature gas chromatography–mass spectrometry (HTGC–MS) with a thermally stable stainless steel capillary column is described as an alternative technique for the analysis of lipophilic compounds (Son *et al*. 2014). In previous studies, it successfully achieved good chromatographic properties for the analysis of lipid molecules including cholesterols (Jung *et al*. 2009, 2010), as well as estrogens with two-phase extractive EOC and sub­sequent PFP derivatization (Moon *et al*. 2011*a*,*b*). Results showed that lower bleeding achieved results in better detectability with a short analytical run compared with a fused silica GC column. The present HTGC–MS-based quantitative cholesterol signatures of 18 sterols including cholesterol, six cholesteryl esters (CEs), three cholesterol precursors and eight oxysterols have been conducted with H-PPT purification and GC separation through a HTGC column separation. All analytes were successfully separated and detected without any interference within a 27-min chromatographic run. The oven temperature was held initially at 260°C for 3 min, ramped to 320°C at 10°C/min, increased to 330°C at 2°C/min (held for 8 min) and finally increased to 380°C at 30°C/min and then held for 3 min. Cholesterol, three cholesterol precursors (desmosterol, lathosterol and lanosterol) and eight OHCs were eluted within 7 min, while six CEs were eluted in the order of the number of carbons in the hydrocarbon chain: cholesteryl laurate (CE 12:0), myristate (CE 14:0), palmitate (CE 16:0), oleate (CE 18:1), linoleate (CE 18:2) and stearate (CE 18:0) (Son *et al*. 2014).

## Hair metabolomics for monitoring potential biomarkers of urological diseases

Although the acute monitoring for drug efficacy is not applicable with hair analysis, hair steroid analysis still gives us the valuable information to confirm the 5α-reductase inhibition after dutasteride administration (Jung *et al*. 2011). With the same pathological events, the biochemical mechanism of male pattern baldness (MPB) was clearly confirmed with hair steroid analysis (Choi *et al*. 2001*b*), and mode of actions of sex steroids in MPB hair samples was differentiated between Caucasian and Korean (Choi *et al*. 2013). In addition to the androgen actions, the cortisol metabolic alteration can be monitored as a biochemical marker of chronic stress, which is an excessive symptom that causes cumulative negative impacts on health outcomes (Lee *et al*. 2015). The detection of cortisol in biological fluids, even saliva, has still been questionable. The increased levels of hair cortisol were observed in childhood obesity, which were also linked to long-term activation of HPA axis (Veldhorst *et al*. 2014), and the risk of cardiovascular disease (Manenschijn *et al*. 2013). Recently, our research team was able to successfully establish the analytical method for the profiling of cholesterol precursors and metabolites (e.g., 7β-hydroxylation of cholesterol).

**Figure 3:**
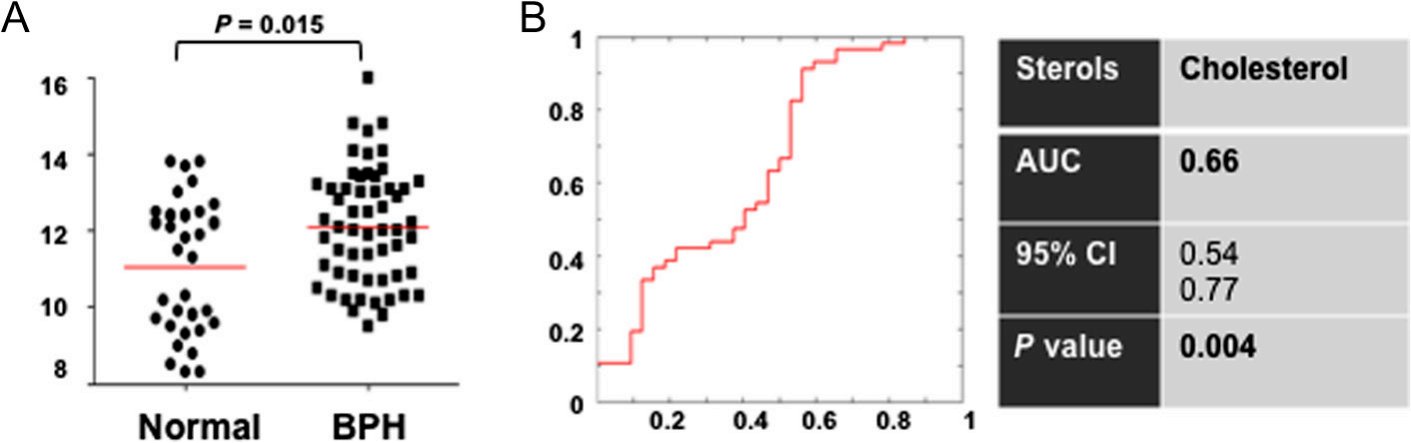
High cholesterol, high sex steroids and hair loss were observed in mice with chronic high-cholesterol diet. Line within the value represents the median.

In the laboratory setting, we have observed that high circulating cholesterol in blood could be associated with high levels of androgens and hair loss in male mice (Fig. 3). Nude mice were grouped (*n* = 5/each group) and fed with high-cholesterol diet or normal chow for 2 months. No weight changes or liver function or dysfunction was observed. Levels of cholesterol and androgen were increased in all mice of high-cholesterol group (five out of five mice). Interestingly, three out of five mice in high-cholesterol group showed hair loss (Fig. 3).

**Figure 4:**
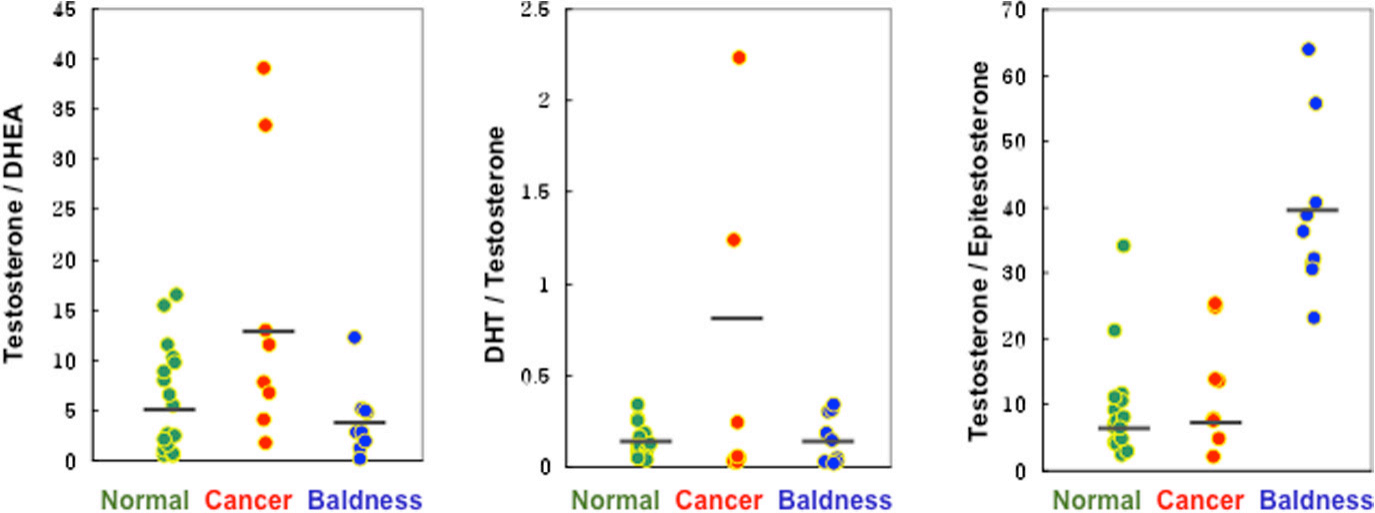
Metabolic ratios of hair androgens in patients with prostate cancer and male pattern baldness compared with healthy control subjects. Line within the value represents the median.

Although increased androgen levels have been associated with both PC and MPB (Demark-Wahnefried *et al*. 1997), no studies have shown an association in hair samples. In establishing a proof of concept, our pilot study showed the increased levels of DHEA, testosterone and DHT in hair samples obtained from both PC and MPB subjects compared with those of age-/sex-matched control subjects. In particular, the metabolic ratios of testosterone:DHEA and DHT:testosterone in PC group tended to increase against the other two groups, whereas a metabolic ration of testosterone:epitestosterone was significantly increased in MPB group (Fig. 4). This is in accordance with our previous findings (Manenschijn *et al*. 2013). These results suggest that the altered metabolic ratios of androgens combined with the higher levels of androgens might serve as the potential biomarkers for PC and MPB.

## Concluding remarks and perspectives

**Figure 5:**
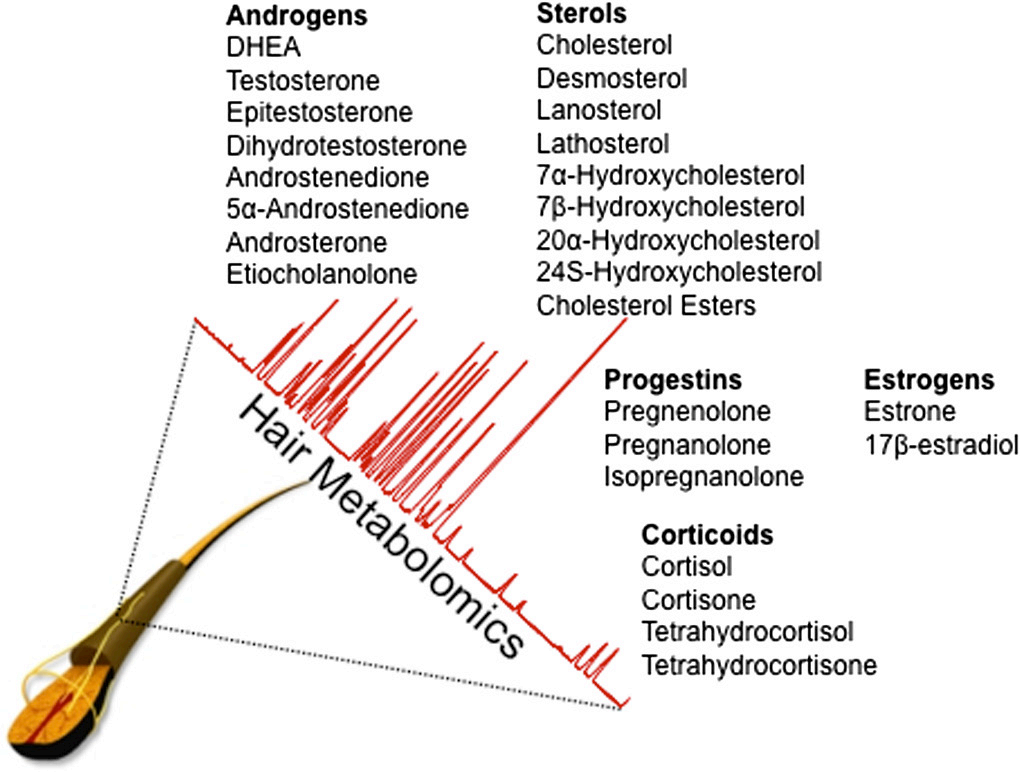
The clinically applicable hair metabolomic approaches to monitor cholesterol and steroid hormone levels in urological diseases.

PC and BPH are characterized by alterations of steroidogenic genes, which are important in synthesis of androgens from cholesterol, or genes converting adrenal androgens to DHT or DHT to inactive metabolites. In this short review article, we summarized these cholesterol and sex steroid metabolic pathways during progression of PC and BPH. Given the evidence derived from our and others’ laboratories, hair metabolomics could be used for monitoring lipoidal hormones, such as cholesterol and sex steroids as well as corticoids. Both synthesis and metabolism of sex steroids with intracrine or paracrine actions are expressed locally in skin, which serve as a target for various steroid hormones including cholesterol (Slominski 2005). Hair as the adnexal structure of the skin contains the entire biochemical apparatus necessary for the production of steroid hormones either from precursors of systemic origin or, alternatively, through the conversion of steroid precursors. Thus, hair metabolomics could therefore be a promising technique for the retrospective assessment of physiological changes in many clinical events including urological diseases. Figure 5 shows important lipid metabolites that our laboratory has successfully established, with the optimized quantitative analysis methods to measure cholesterol and sex steroid hormones for monitoring, using hair metabolomics.

## Declaration of interest

The authors declare that there is no conflict of interest that could be perceived as prejudicing the impartiality of this review.

## Funding

The authors acknowledge the support from the Korea Institute of Science and Technology Institutional Program (Project No. 2E26110 (to M H C)), National Institutes of Health grants (1U01DK103260, 1R01DK100974, U24 DK097154, NIH NCATS UCLA CTSI UL1TR000124 (to J K)), Department of Defense grants (PR140285 (to J K)), Centers for Disease Control and Prevention (1U01DP006079 (to J K)), IMAGINE NO IC Research Grant, the Steven Spielberg Discovery Fund in Prostate Cancer Research Career Development Award, and U.S. – Egypt Science and Technology Development Fund by the National Academies of Sciences, Engineering, and Medicine. J K is the former recipient of Interstitial Cystitis Association Pilot Grant, a Fishbein Family IC Research Grant, New York Academy of Medicine, and Boston Children’s Hospital Faculty Development.

## Authors’ contribution statement

M H C and J K designed the study, led obtaining funding and overviewed the literature analysis and drafting the manuscript. J Y M performed the analysis of references and assisted in writing the manuscript. All authors read and approved the final manuscript.

## Availability of data and materials

All the data supporting the findings here is contained within the manuscript.
